# Dealing with Context through Action-Oriented Predictive Processing

**DOI:** 10.3389/fpsyg.2012.00421

**Published:** 2012-10-19

**Authors:** Julian Kiverstein, Erik Rietveld

**Affiliations:** ^1^Department of Philosophy, Institute of Logic, Language and Computation, University of AmsterdamAmsterdam, Netherlands; ^2^Cognitive Science Center Amsterdam, University of AmsterdamAmsterdam, Netherlands; ^3^Department of Psychiatry, Academic Medical Center, University of AmsterdamAmsterdam, Netherlands

Contemporary neuroscience seems to be undergoing an intriguing pragmatist turn (Engel, 2010). Clark’s action-oriented account of the predictive brain offers an original and interesting take on pragmatist neuroscience. The idea that cognition is for action is of course central to the embodied program of research in cognitive science. Clark’s account of the predictive brain provides a promising proposal about how the brain might contribute to embodied cognition. However within the predictive coding framework there is also recognition of the important role played by context in everyday action over multiple time scales (Kiebel et al., 2008). We will argue that the context sensitivity of action-oriented processing is not adequately recognized in Clark’s target article. The ecological notion of a niche (e.g., Gibson, 1979) is for instance central in Friston (2011) account of embodied cognition, but we find it curiously absent in the account Clark gives of action.

An animal’s eco-niche is made up of affordances (Gibson, 1979; Chemero, 2003) or possibilities for action provided to the animal by an environment – by the substances, surfaces, objects, and other living creatures that surround it. Importantly, affordances are always perceived in the context of many other affordances available in the niche (cf. Cisek and Kalaska, 2010). The landscape of affordances on offer in a given environment is exceptionally rich (particularly in the case of humans) and is responded to in a way that takes both the broad environmental context (e.g., the current place: restaurant or swimming pool) and the internal state (including for instance metabolic needs) of the individual agent into account. The “behavior setting” (Barker, 1968; Heft, 2001) is an excellent predictor of what action possibilities will show up for a person as relevant: when we are in a restaurant the possibility of calling a waiter is relevant but not when we are in a supermarket. Moreover, affordances can solicit activity because they are bodily *potentiating*. A relevant affordance can generate bodily “action readiness” (Frijda, 1986, 2007), that is, the readiness of the affordance-related ability (Rietveld, 2008). Just as our hand prepares itself to grasp a cup when we make a reaching movement for a cup, so also can being in a particular behavior setting like a restaurant make certain action possibilities more salient to us. In both cases we find ourselves bodily ready to deal with particular aspects of a familiar environment. Once we recognize the richness of the landscape of affordances in any given context of activity, this generates parsimonious answers to old questions about the relation between mind and world (Rietveld and Kiverstein, under review), but it also opens up a host of difficult questions for cognitive scientists. How is it possible that in any given situation an agent is able to hone in on the action possibilities that are relevant while also remaining sensitive to other action possibilities of potential relevance? In a dynamic environment in which our situation is continuously undergoing change, how do we take new information into account in such a way as to produce actions that fit with the behavior setting in which we are embedded?

Action-oriented predictive processing as described by Clark is, we are sure, an important part of the answer to these hard questions (Kiverstein, 2012). Part of the reason why it is well equipped to answer these difficult questions is because it provides us with an account of neural processing on which perception, action, and cognition are as Clark puts it “deeply unified.” A challenge that remains for Clark’s action-oriented theory of the brain is to account for how our skillful unreflective actions involve the integration of the whole system of brain, body, and the landscape of available affordances in which we are situated.

The phenomenology of skillful unreflective action in everyday life provides us with a clue here. In a skilled individual’s responsiveness to a field of relevant affordances (Rietveld et al., 2012; BBS) it is not only perception, action, and cognition that are profoundly unified, but also affect and the eco-niche or environment. Thanks to our skill and expertise often we find ourselves in a situation in which affect makes it immediately obvious to us how we should respond. Take the case of responsiveness to social affordances: a friend’s sad face invites comforting behavior, a colleague at a coffee machine affords a conversation, and the extended hand of a visitor solicits a handshake. Affect plays a crucial role in preparing us to act in these cases: it signals which possibilities for action in a situation matter to us in sense of being relevant to us given our interests and needs. Thus it contributes toward an agent’s selective perception of and responsiveness to the action possibilities available in a given situation. Affect can also play a role in keeping us apprised of how well an action is going when we are in the flow of performing an action (see Rietveld, 2008). “The context” is not a static pre-given: What is in the foreground and what is in the background for us constantly shifts, depending both on what happens in the environment, and on our current concerns. In other words, affect contributes to the structuring of the field of relevant affordances in the particular situation.

No doubt this can partly be explained in terms of action-oriented predictive processing and the cascades of generative models that are able to successfully predict how to bring about the sensory consequences the agent anticipates. However we would argue that a challenge for Clark remains to integrate this neural level explanation with what the phenomenology of everyday agency tells us. More precisely, we must account for how neural processing is always taking place within a particular situation in which specific possibilities of action stand out from a larger landscape of affordances as soliciting or motivating for the agent.
